# Using video-based examiner score comparison and adjustment (VESCA) to compare the influence of examiners at different sites in a distributed objective structured clinical exam (OSCE)

**DOI:** 10.1186/s12909-023-04774-4

**Published:** 2023-10-26

**Authors:** Peter Yeates, Adriano Maluf, Natalie Cope, Gareth McCray, Stuart McBain, Dominic Beardow, Richard Fuller, Robert Bob McKinley

**Affiliations:** 1 School of Medicine, Keele University, David Weatherall Building, Keele, Staffordshire, ST5 5BG UK; 2 Fairfield General Hospital, Northern Care Alliance NHS Foundation Trust, Bury, Greater Manchester, UK; 3 Christie Education, Christie Hospitals NHS Foundation Trust, Manchester , UK

**Keywords:** OSCE, Assessment, Equivalence, Examiner-Cohorts, Distributed Assessment

## Abstract

**Purpose:**

Ensuring equivalence of examiners’ judgements within distributed objective structured clinical exams (OSCEs) is key to both fairness and validity but is hampered by lack of cross-over in the performances which different groups of examiners observe. This study develops a novel method called Video-based Examiner Score Comparison and Adjustment (VESCA) using it to compare examiners scoring from different OSCE sites for the first time.

**Materials/ methods:**

Within a summative 16 station OSCE, volunteer students were videoed on each station and all examiners invited to score station-specific comparator videos in addition to usual student scoring. Linkage provided through the video-scores enabled use of Many Facet Rasch Modelling (MFRM) to compare 1/ examiner-cohort and 2/ site effects on students’ scores.

**Results:**

Examiner-cohorts varied by 6.9% in the overall score allocated to students of the same ability. Whilst only a tiny difference was apparent between sites, examiner-cohort variability was greater in one site than the other. Adjusting student scores produced a median change in rank position of 6 places (0.48 deciles), however 26.9% of students changed their rank position by at least 1 decile. By contrast, only 1 student’s pass/fail classification was altered by score adjustment.

**Conclusions:**

Whilst comparatively limited examiner participation rates may limit interpretation of score adjustment in this instance, this study demonstrates the feasibility of using VESCA for quality assurance purposes in large scale distributed OSCEs.

## Background

Although contemporary views of assessment have evolved considerably over the last decade [1], Objective Structured Clinical Exams [2] remain a cornerstone of many programs of assessment programs because of their ability to assess students’ performances on a structured range of blueprinted tasks under formally observed conditions [3] and because they aim to ensure equivalence of standards [4] for all students who are assessed. The latter is important as it is critical to both the ability to reassure patients that consistent standards have been met and to ensure that students are judged according to a comparable, and therefore fair, standard.

Being able to demonstrate equivalence of OSCE exams is, consequently, a key component of the chain of validity [5] on which resulting assessment decisions are based. In contrast to the original conception of OSCEs [6], candidate numbers in most institutions require the use of multiple parallel versions of (ostensibly) the same OSCE ‘diet’, with different groups of examiners and students in each parallel form of the test. As a result, it becomes necessary for each separate group of examiners to collectively judge to the same standard in order to ensure equivalence across these multiple parallel forms of the same test, otherwise whether or not a student passes or fails may depend on the parallel form of the test to which they were allocated, rather than solely on their ability per se. Whilst the meaning of fairness can be debated [7], few educators would find it reasonable that a students’ outcome could be determined by their allocation to a circuit or location within the exam.

Ensuring the equivalence of different groups of examiners (or “examiner-cohorts” [8]) is difficult; conventional psychometric analyses of reliability do not readily provide parameters which describe examiner-cohorts effects. One limitation in determining equivalence across different examiner-cohorts is that students and examiners are typically “fully-nested”, that is, there is no overlap between the students examined by different groups of examiners, so there is no means to directly compare scoring without making strong assumptions about equivalence of students’ performance in each parallel form of the test.

Owing to these methodological difficulties, research on the influence of different examiner-cohorts has been limited, but a few studies are informative. Floreck et al. [9] found up to 0.0-15.7% of score variance in an OSCE could be attributed to different sites. Consistently Sebok et al. [10] found raters at different sites contribute 2.0–17% of variance. In each instance we can see that whilst variations between groups of examiners was not ubiquitous, it occurred in some instances and had the potential importantly influence outcomes (e.g., graduation or progression) for some candidates.

More recently, Yeates et al. have developed a method called “Video-based Examiner Score Comparison and Adjustment” (VESCA) [11, 12], which aims to provide a means to compare otherwise unlinked groups of examiners in OSCE exams. VESCA involves a 3-stage process, based on 1/ filming a small subset of candidates on each station of the OSCE in order to provide exemplar video-based performances, 2/ asking all examiners to score a number of station-specific (i.e. from the station they have just examined) video-based performances in addition to scoring live candidates. Each different group of examiners collectively score the same video performances. 3/ using the overlap created by examiners’ video scores to link the otherwise unlinked examiner-cohorts in statistical analyses and compare their effects.

Yeates et al. recent studies showed differences in the standard of judgement between examiner cohorts of up to 5.7% [11] and 7.1% [12] respectively. On both occasions, adjusting for these differences would have altered pass/fail decisions for a subset of candidates. Their approach varied between the studies, with interim development in the filming approach [13], an increase in the number of videos examiners were asked to score, and variations in the method of video scoring, including trialing an approach to internet-based video scoring by examiners [12]. Subsequent *post hoc* analyses of data from the latter study have examined for the presence of substantial biasing influences in the form of contrast or DRIFT effects [14]. This study found no evidence of contrast effects in these data and only slight evidence to support DRIFT effects. As both of these effects could potentially bias VESCA’s estimates, their absence is reassuring. Further *post hoc* analyses have examined the impact on score adjustments of 1/ the number of linking videos examiners were asked to score and 2/ examiner participation rates [15]. It concluded that very similar score adjustments would be expected from either 3 linking videos (as opposed to 4) or from 60 to 70% examiner participation, but that score adjustments derived from fewer linking videos or fewer participating examiners would produce larger discrepancies in score adjustments.

Consequently, this programme of research has provided a growing degree of insight into the utility of VESCA as a means to compare the influence of examiner-cohorts on students’ scoring within OSCEs. Nonetheless, whilst the stated intent and the greatest theoretical benefit of VESCA is in comparing examiner-cohorts across different locations within a distributed OSCE exam, each of these prior studies has used VESCA to compare examiner-cohorts within the same geographic location. As it is critical to developing the utility of VESCA before it can be used in practice, this study aimed (for the first time) to use VESCA to compare the scoring tendencies of different examiner-cohorts based in different geographical locations within an OSCE, by addressing the following research questions:

1. How does the stringency / leniency of different examiner cohorts compare within a multi-site OSCE?
2. How does the stringency / leniency of examiners compare between different OSCE sites within a multi-site OSCE?
3. What are the relative magnitudes of any within vs. between site differences in examiners stringency / leniency?
4. What is the (theoretical) impact of adjusting students’ scores for any observed differences in examiner-cohort stringency/leniency on their overall score, pass/fail outcome and rank position within the OSCE?

## Methods

### Overview

We employed the VESCA methodology as described by Yeates et al. [12] to compare examiner-cohort effects across 2 sites within a distributed OSCE exam. All participating examiners scored videos after the OSCE via a secure website. Video scores and live score data were amalgamated to address the research questions, by using Many Facet Rasch Analysis.

### Assessment context

We used VESCA within the context of the Year 4 undergraduate OSCE at Keele University School of Medicine, in June 2021. This was a summative OSCE, which contributed substantially to students’ progression into the last year of the course. It comprised 16 × 10-minute stations, which each integrated a range of skills such as history taking, physical examination, patient counselling, practical procedural skills, clinical reasoning, investigation and management planning. Stations comprised simulated clinical scenarios depicted by trained simulated patients and covered a broad range of clinical disciplines including internal medicine, surgery, emergency medicine, general practice, obstetrics and gynaecology and paediatrics. The OSCE was conducted face to face, but usual OSCE practices were adapted due to the COVID-19 pandemic to exclude real patients (healthy actors were used instead). All participants wore appropriate personal protective equipment including face masks. Examiners were clinicians who had previously received training (including generic video benchmarking) on OSCE conduct. Examiners scored performances using Keele’s GeCoS domain-based rating scale [16], which elicited proficiency scores [1–4] on 5 station-relevant domains (for example “history content”, “building and maintaining the relationship”, “clinical reasoning”) plus a global score out of 7 to give each station a maximum score out of 27.

The OSCE was run across 2 separate locations, approximately 37 miles apart. These locations are both hospital sites and both routinely host approximately 50% of each cohort of year 4 Keele University’s medical students for their clinical placements throughout the year of study. One site is a tertiary referral centre, whilst the other is a secondary referral centre. One site serves a comparatively urban and deprived population whilst the other serves a comparatively rural and affluent population. Within both sites there were 2 parallel circuits (or tracks) of the OSCE, and in both there were separate morning and afternoon sessions between which examiners frequently changed. As a result, there were 8 comparatively unique groups of examiners (morning and afternoon x2 circuits, x2 sites). The design was fully nested with no crossover between the students seen by different examiner-cohorts, with most examiners examining at the site where they usually worked.

### Population, sampling and recruitment

Our study population was students and examiners participating in the OSCE. We used whole-group email to recruit a volunteer sample of students and examiners to be videoed, aiming to recruit at least 8 students and 16 examiners (included in order of volunteering). For pragmatic reasons, all filming was conducted at one site, so only examiners and students from that site were eligible. There were no other exclusion criteria. Simulated patients were recruited by email from Keele School of Medicine’s database of simulated patients.

### Ethics

All participation by students and examiners in the research procedures was voluntary. All participants were greater than 18 years of age and provided informed consent. Participants had the right to withdraw. Data were treated confidentiality and pseudonymized once feasible. All methods were carried out in accordance with relevant guidelines and regulations. Ethical approval for the study was granted by Keele University Ethics Committee (reference MH-190,102).

### VESCA research procedures

Videoing: Volunteer students and simulated patients were filmed on each station of the OSCE using two wall-mounted CCTV cameras (ReoLink 423) which were moved to appropriate positions for the station content, using the principles developed by Yeates et al. [13]. Audio was recorded using boundary microphones. Participating students were filmed on all 16 stations within the OSCE. Examiners were not deliberately filmed but sometimes featured in the background of videos. Video footage was processed by an audio-visual technician to provide a blend of wide-angle and close-up views of students’ performances, resulting in 8 videos of each station. Where detailed views of physical items were required (for example to show students’ labelling of specimen tubes / details of an ECG), these were collected between students by researchers, numbered and either photographed or scanned. The first 4 videos of each station which showed unobstructed pictures and adequate sound were selected for use in subsequent video scoring. Videos were available 24 h after the OSCE was completed.

Video scoring: The 4 selected videos for each station were uploaded to a secure web-survey system (Keele’s Faculty of Medicine and Health Survey System) which is built on the open-source websurvey system Lime Survey (LimeSurvey GmbH) [17]. Once videos were available and uploaded, all examiners who participated in the OSCE (all examiner-cohorts at both sites) were invited via email to score videos from their station as part of a research project. Examiners had received email advertisements for the study in the weeks running up to the OSCE so were aware that these invitations would be sent. The web-based survey system presented examiners with a participant information sheet, obtained consent and played a test video to ensure their audio and picture were adequate. Next examiners were streamed into a portion of the survey which was specific to the station they had examined. There they were presented with the same examiner information they had been given during the exam (including marking criteria), before being presented with four videos of students performing on the station they had examined. They were asked to observe, score and provide written feedback to each performance sequentially. The web-survey system collected scores according to the specific scoring domains for each station. Following completion of video scoring examiners were provided brief de-briefing information.

For each station, all participating examiners scored the same video performances. Examiners who had scored the videoed performances live were also invited to score videos so that live and video scoring could be compared. Examiners who examined different stations (for example on different days) were invited to score each of the stations they had examined. Reminder emails were sent via the survey system to examiners who had not yet participated. All invitations included an option to decline to participate and opt out of future invitations.

Analysis: Scores which examiners allocated to video performances were aligned with the score data from the OSCE to create one dataset. To address the study research questions, data were analysed with Many Facet Rash Measurement (MFRM), using FACETS v3.83.6 [18]. We performed a number of analyses to determine the suitability of the data for MFRM. Firstly, we examined examiner participation rates, comparing these across different examiner-cohorts. Secondly, we compared the distribution of scores within video performances with the distribution of overall performances. Next we used a Bland-Altmann plot [19], using the package ‘BlandAltmanLeh’ [20] in R to compare scores given to the same performances by the subset of examiners who examined them under both “live” and “video” condition, to determine whether there were any differences in scores added to video and live performances. Following this, we examined Mean Square Infit and Outfit parameters provided by FACETS software to determine whether data fit the Rasch model, using the fit parameters suggested by Linacre [21], i.e. that Mean Square values between 0.5 and 1.5 indicate productive fit for measurement. Finally, as MFRM makes strong assumptions of unidimensionality, we performed Principal Components Analysis (PCA) of model residuals to seek any evidence of additional (and therefore distorting) dimensions within the data, using base R [22].

To address our principal research question, we used a four facet Rasch model in FACETs, with a dependent variable of score (out of 27 scale points), with facets of: Student (ability), station (difficulty), site (site effect), and examiner-cohort (stringency). We used output of this analysis to compare examiner-cohort effects, site effects, and examiner-cohort effects within sites. We calculated students score adjustments by subtracting the observed from the adjusted score for each student and calculating descriptive data.

To determine the influence of score adjustment on pass/fail categorization, we obtained the cut score using the institution’s standard approach of borderline regression [23] + 1 additional SEM. We then compared student observed (unadjusted scores) and adjusted scores with the cut score to determine which students passed and failed in each condition. We did not consider impact on conjunctive passing rules as the MFRM only adjusts overall (rather than station level) scores.

To determine influence of score adjustment on rank, we firstly order students by unadjusted score and noted their rank position, then ordered by adjusted scores and noted their rank position, and then computed the change in rank by subtracting the unadjusted rank from the adjusted rank for each student and calculating descriptive statistics.

## Results

### Descriptive data and participation rates

Data were available for all 126 students who were assessed within the OSCE. Students unadjusted average scores (i.e. their average across all 16 stations) ranged from 15.5 out of 27 (57.4%) to 24.1 out of 27 (89.3%), and were normally distributed with a mean of 20.9 and SD of 1.6. Mean scores varied by station, from 18.7 (69.2%, the hardest stationi) to 22.5 (83.5%, the easiest station), thereby showing the anticipated range of station difficulty. The Cronbach alpha for the data overall was 0.76, with Corresponding Cronbach’s alpha of 0.73 for site 1 and 0.79 for site 2.

Students who volunteered to be filmed had unadjusted average scores ranging from 17.0 (62.9%) to 22.3 (82.6%) with a median of 20.6. Consequently, videoed students’ average ability range constituted 61% of the range of total student ability. Scores allocated to individual video performances by examiners ranged from 9 (33.3%) to 27 (100.0%) with a median of 20 (74.1%), thereby covering a wider range of levels of performance.

Examiners scored comparison videos on 242 out of 512 available opportunities, giving an overall examiner participation rate of 47.3%. Video scoring rates varied by station, ranging from scoring completed on 0 out of 32 (0.0%) eligible occasions for station 16, to scores provided on 28 out of 32 (87.5%) eligible occasions for station 10. Examiner participation rates also varied by site, with videos scored on 80 out of 256 (31.2%) eligible occasions by examiners from site 1, whilst videos were scored on 162 out of 256 (63.3%) eligible occasions by examiners from site 2.

Given these examiners participation rates, we further examined the degree of score linkage which the video scores had achieved between the 2 OSCE sites. Forty-six out of sixty-four comparison videos (71.9%) were scored by at least one examiner from each site. Whilst no formal method exists to determine linkage adequacy, this is expected to be sufficient for the analyses we performed. See Table 1 for demonstration of the linkage pattern produced by video scores. Score allocated to videos comprised 11% of the total dataset.

**Table 1:** Linkage Pattern

	Stations															
	1	2	3	4	5	6	7	8	9	10	11	12	13	14	15	16
1st video for this station	Number of examiners from each site who scored each performance															
site 1	4	2	1	0	0	2	2	1	1	3	1	0	2	1	0	0
site 2	1	2	2	4	4	3	3	3	2	4	0	4	1	3	2	0
2nd video for this station																
site 1	4	2	1	0	0	2	2	1	1	3	1	0	2	1	0	0
site 2	1	2	4	4	4	3	3	3	2	4	2	4	1	3	2	0
3rd video for this station																
site 1	4	2	1	0	0	2	2	1	1	3	1	0	2	1	0	0
site 2	1	2	4	4	4	3	3	3	2	4	0	4	1	3	2	0
4th video for this station																
site 1	4	2	1	0	0	2	2	1	1	3	1	0	2	1	0	0
site 2	1	2	4	4	4	3	3	3	2	4	2	4	1	3	2	0

### Adequacy of data for many facet rasch models

**Fig. 1:**
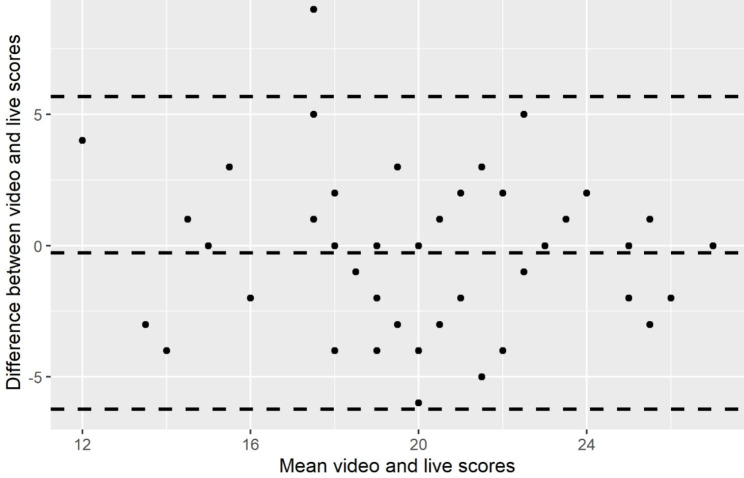
Bland Altman Plot comparing live and video scores

The Bland-Altman plot which compared the scores given by the same examiners to the same performances in both “live” and video formats had a mean and 95%Cis of -0.28 (-6.24–5.67), indicating that there was no significant difference between live and video scoring (see Fig. 1).

The PCA of models residuals, used to examine dimensionality of the data, showed that all eigenvalues were > -2, a commonly used cut-off for indicating that variance is at a random level [24], As a result, there was no indication that data breached unidimensional assumptions.

Data generally showed good fit to the Many Facet Rasch Model. Mean Square Init and Outfit values were 0.5–1.5 for all 16 stations, all 8 examiner-cohorts and for both sites. Four out of the 126 students (3.2%) showed overfit to the Many Facet Rasch Model (MnSq < 0.5). Eleven students showed underfit to the model (MnSq > 1.5). Of these, 9 showed mild underfit (MnSq 1.5–1.7, Z std < 2.0). Two students showed greater underfit (MnSq 2.0-2.3, Z std > 2.0). As these students’ data had the potential to distort the model, we removed them and recalculated the model. This resulted in a median alteration of students’ score adjustments of 0.05% of the assessment scale. On this basis we determined that these data were not distorting the model and proceeded with the full dataset.

### Main research question

1. How does the stringency / leniency of different examiner cohorts compare within a multi-site OSCE?

**Fig. 2:**
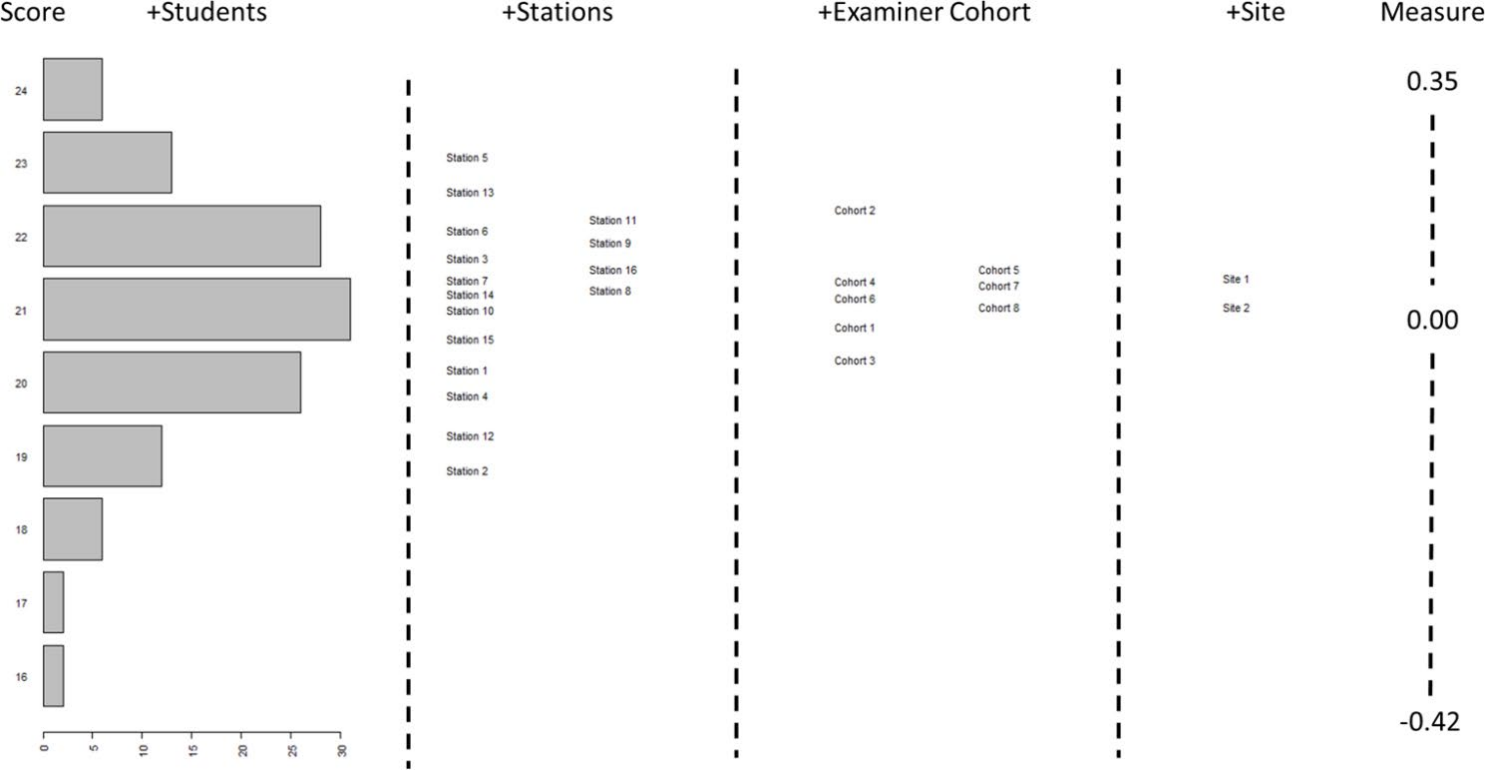
Wright Map showing relative influence of items within all four facets: students, stations, examiner-cohorts, and site

Observed (unadjusted) average values for the 8 examiner-cohorts ranged from 20.23 (74.9%) for examiner cohort 5 to 21.39 (79.2%) for examiner cohort 2. Model-derived parameters (which make use of the linkage provided by scores given to the comparison videos to compare the effects of these examiner cohorts) produced a different rank ordering of examiner-cohorts, with the lowest adjusted score of 20.28 (75.1%) for examiner cohort 3, and the highest adjusted score of 22.13 (82.0%) for examiner cohort 2, giving a difference of 1.85 (6.9%). Notably, these scores are the model estimates of the score that a student *of the same ability* would have received from these different examiner cohorts. These data are illustrated in Fig. 2.

Whilst FACETs output does not provide a formal test of statistical significance, considering these data on the logit measurement scale enables them to be appreciated relative to the model standard error. Examiner-cohort 3 had a logit measure value of 0.28 logits and examiner-cohort 2 had a logit measure value of 0.47. The model standard error was 0.02, therefore the difference between the highest and lowest examiner-cohort (0.47 − 0.28 = 0.19 represents 9.5 multiples of the model standard error making the differences very unlikely to be due to measurement error.

As well as demonstrating these differences, model-adjusted parameters changed the rank order of examiner-cohorts, with unadjusted scores suggesting an order from most stringent to most lenient of examiner cohorts: 5, 7,4, 3, 6, 8, 1, 2; whereas the model-adjusted scores suggested an order from most stringent to most lenient of examiner cohorts: 3, 1, 8, 6, 7, 4, 5,2.

2. How does the stringency / leniency of examiners compare between different OSCE sites within a multi-site OSCE?

Observed (unadjusted) average values for the 2 OSCE sites were 20.97 (77.7%) for site 1 and 20.73 (76.8%) for site 2, a difference of 0.24 (0.9%). Model derived estimates for site differences suggested a minimally larger difference between the OSCE sites, with parameters of 21.28 (78.8%) for site 1 and 20.92 (77.5%) for site 2, giving a difference of 0.36 (1.3%). Corresponding logit measure values for OSCE site 1 was 0.02 logits and for OSCE site 2 was − 0.02 logits with a model standard error of 0.01. Consequently, the difference between these parameters of 0.04 logits is 4 times the model standard error (0.04/0.01 = 4) and therefore this very small difference may still be considered statistically significant.

3. What are the relative magnitudes of any within vs. between site differences in examiners stringency / leniency?

**Fig. 3:**
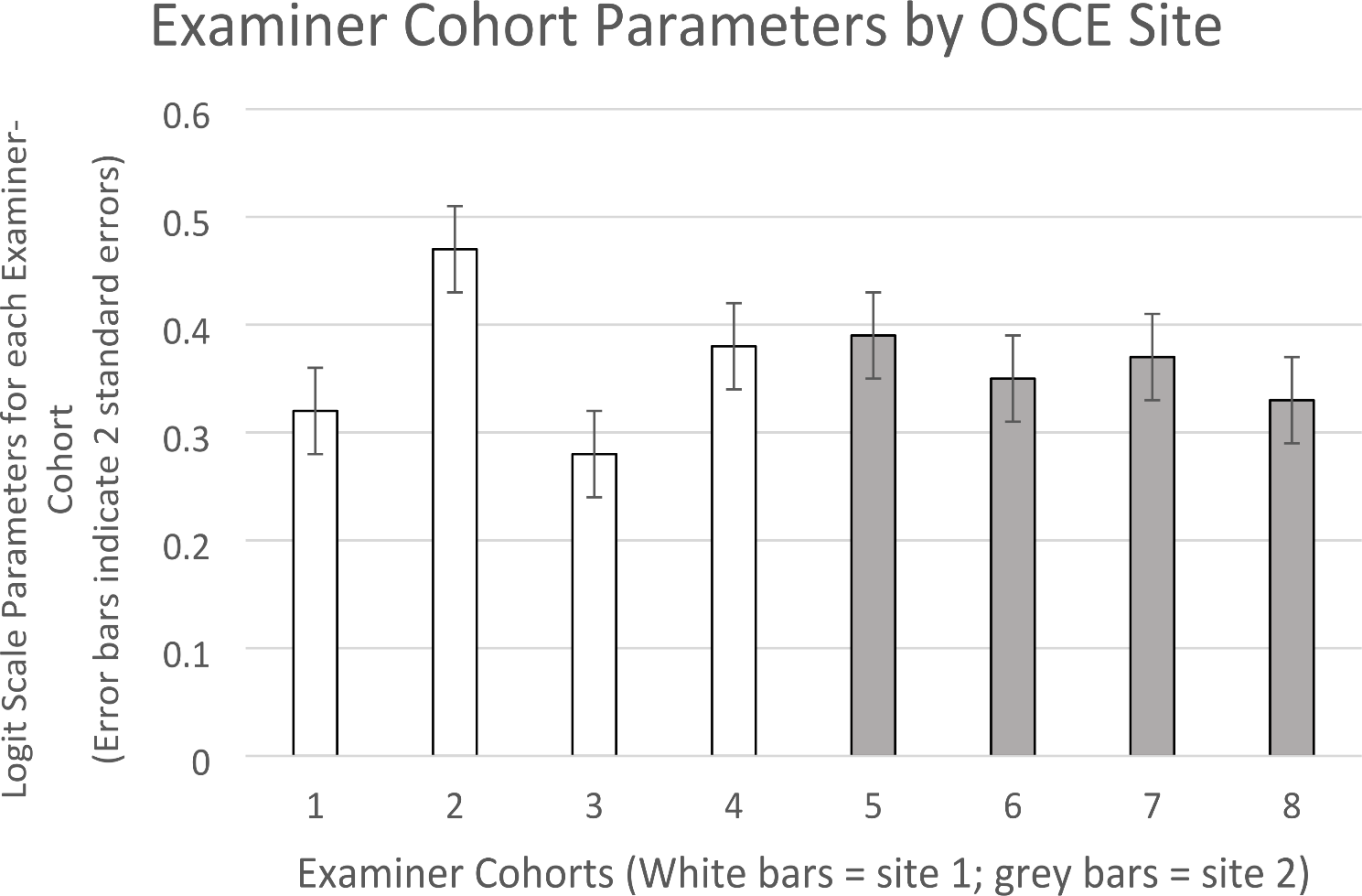
Examiner-Cohort Parameters by Site

Organising examiners-cohorts by sites showed that *within* site variation was greater at both sites then *between* site variation. Moreover, the magnitude of variation between examiner cohorts varied between sites. For site one, model-adjusted score parameters ranged from 20.28 (75.1%) for examiner-cohort 3 to 22.13 (82.0%) for examiner-cohort 2, a difference of 1.85 score points (6.9%). These were the most stringent and the most lenient examiner cohorts in the OSCE, and as explained earlier, are arguably statistically significantly different based on comparison of logit measure values in comparison to the model standard error. By contrast, for site two, model-adjusted score parameters ranged from 20.77 (77.0%) for examiner cohort 8 to 21.39 (79.2%) for examiner-cohort 5, a difference of 0.62 score points (2.3%). Moreover, examination of the same logit-scale measure values relative to the model standard error suggests that these differences were not statistically significant (examiner cohort 8 = 0.33 logits; examiner cohort 5 = 0.39 logits, difference 0.06 logits, model SE 0.02 logits, therefore difference = 3x model standard error). These parameters are displayed graphically in Fig. 3.

4. How does adjusting students’ scores for any observed differences alter their score, pass/fail categorization or rank position within the OSCE?

**Fig. 4:**
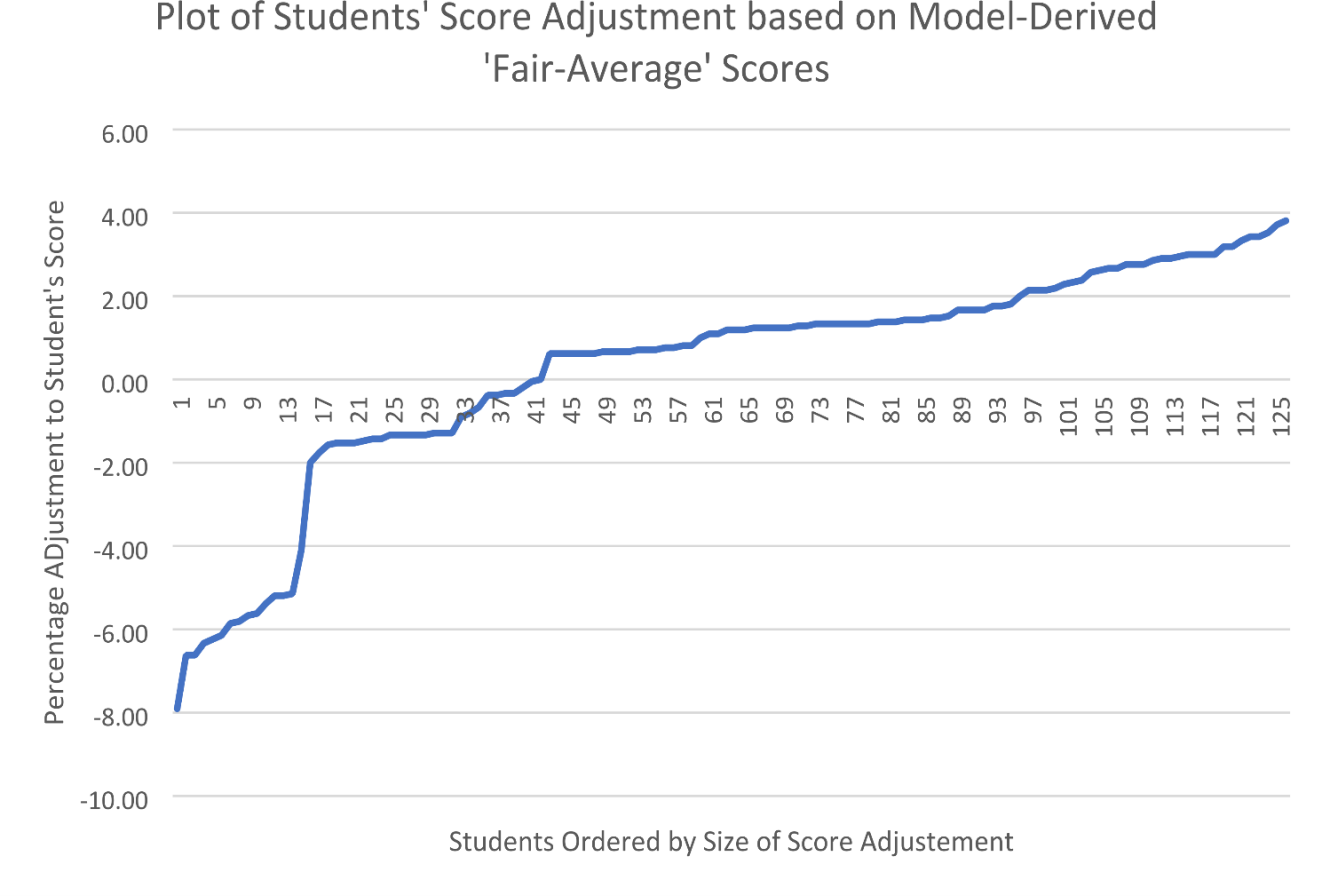
Plot of Students’ score adjustments

Although video scores only compare examiners scoring directly for a limited subset of students, the linkage that this creates enables the Many Facet Rasch Model to estimate adjusted scores for all students (including those who weren’t videoed). Model-based ‘fair scores’ for students’ average overall performance differed from the corresponding observed scores and would result in adjustment to students’ scores if these were adopted. Notably, the model-derived scores aim to correct for differences in the stringency of the examiner cohort which each student met. Students’ score adjustments (fair average score – observed average score) ranged from − 1.66 scale points (-7.9%) to + 0.80 scale points (3.8%). Fourteen students’ scores (11.1%) were adjusted downward by at least 5% of the scale (in response to lenient examiners) whilst twelve students’ scores (9.5%) were adjusted upwards by at least 3% (in response to stringent examiners. Sixty-six students (52%) received score adjustments (either up or down) of < ± 1.5%. The median score adjustment (regardless of whether up or down) was 0.31 scale points (1.1%). The distribution of score adjustments suggested by the model are shown in Fig. 4.

The cut score for the test was 16.38 out of 27. Score adjustments had a minimal impact on students’ pass/fail categorization. In the observed (unadjusted) scores, 124 students (98.4%) passed the OSCE; in the adjusted scores 123 students (97.6%) of students passed. 1 student (0.8%) changed from pass to fail whilst no students changed classification from fail to pass.

Scores adjustments influenced students’ rank position within the OSCE. The largest increase in rank was 20 rank places (1.6 deciles), whilst the largest decrease in rank was − 44 rank positions (-3.5 deciles). Four students (3.2%) changed rank position by more than 3 deciles. Nineteen students (15.0%) increased their rank by at least 1 decile (i.e. > 12.6 rank places), whilst a further 15 students (11.9%) decreased their rank position by at least 1 decile. The median change in rank (regardless of whether rank increased or decreased) was 6 rank positions (0.48 deciles).

## Discussion

### Summary of findings

In this study we have again shown that VESCA procedures can be used to provide estimates of examiner-cohort effects in a distributed OSCE, this time comparing across different geographical locations for the first time. Examiner participation rates were lower than in previous studies, ranging from 31 to 62% by site. Results showed that examiner cohorts varied in their stringency / leniency with a 6.9% difference between estimates of the scores the highest and lowest examiner-cohorts would give to a student of the same ability. There was minimal overall difference between the stringency / leniency of examiners in each site (1.3%) but the apparent variation in stringency between groups of examiners was much greater in site 1 than in site 2. Adjusting students’ scores for these differences produced a substantial alteration for a subset of students, resulting in greater than 1 decile change in rank for 26.9% of students and a 3 decile change in rank position for 3.2% of students. By contrast, only 1 student (0.8%) changed their pass/fail categorization as a result of score adjustment.

### Theoretical considerations

Taken at face value, the differing estimates of leniency / stringency shown by different-examiner cohorts in this study offer some challenge to the equivalence of the OSCE for students in different examiner-cohorts. Whilst the validity argument for an OSCE relies on an evidentiary chain which includes its blueprint, content, station design, scoring approach and faculty and simulated patient development efforts [25], few would argue that the group of examiners to which a student is allocated should influence their score. Moreover, the size of change in score and the change in rank position for some students would raise questions about the degree to which observed scores reflect their performance if, indeed, the score adjustments suggested by these data can be validated.

Notably, score adjustment produced very different impacts on students pass/fail categorization and on their rank. This is likely to be attributable to two factors: firstly, in this instance very few students were near to the pass/fail boundary, so despite many students receiving notable adjustments to their scores this did not influence their classification because their observed scores were sufficiently far from the cut score. Secondly, students’ scores were relatively tightly distributed, so these adjustments were sufficient to produce changes in rank which were sometimes substantial. Two points are theoretically important in relation to this. Firstly, whilst the impact on pass/fail categorization was limited in this instance, the same score adjustments would have had a substantial impact on pass/fail categorization in an OSCE with a higher observed (unadjusted) failure rate, such as was demonstrated in Yeates et al’ 2021 paper [12]. Secondly, consideration of the impact on students’ rank is important as OSCEs often contribute to students’ academic rank within institutions.

One of the attractions of the VESCA methodology is its ability to link groups of examiners through comparative scoring of a limited subset of video performances and then use this linkage to extrapolate beyond the crossed data to instances which were not videoed. Consequently, it is critical to the validity of the technique to understand how effectively the comparative video scores enable the model to estimate effects for examiner cohorts, sites and students within a single frame of reference. As stated in prior research [12] this question probably requires statistical simulation to determine the likelihood of VESCA produce accurate estimates under various circumstances and we continue to advocate for the need for such research before VESCA is used in practice.

Nonetheless, some existing literature enables us to reflect on the likely trustworthiness of these data. Examiner participation was lower in this study than in prior uses of VESCA (48% overall vs. 73.1% [11] and 76.0% [12]). Recent work by Yeates et al. [15] examined the influence of fewer examiners on the size of score adjustment that each student received. Working with a limited number of permutations, they found that score adjustments were highly correlated when 76%, 70% and 60% of examiners participated, but score adjustments derived from 50% of participating examiner showed lower correlations with those derived from a higher proportion of participating examiners (rho = 0.29–0.93). As a result, we may presume that score adjustments in our study may have differed had a higher proportion of examiners opted to take part. More generally, recent work by Homer [26] suggests that correcting for examiner stringency (i.e. score adjustment) can falsely inflate passing rates. This further illustrates the need to understand the validity of statistical correction for examiner stringency under a range of circumstances.

The observation that the difference between sites was very small is likely to help to reassure participants in the assessment. It is notable that variability in examiner-cohorts’ stringency differed between sites. Two different mechanisms may plausibly explain this observation. Firstly, it may be that examiners in site 1 had a less shared frame of reference and so varied more in the standard of their judgements. If this were the case, then VESCA would illustrate the need for greater faculty development efforts with this site. Alternatively, it could be that the lower examiner participation rates in site 1 inflated variability in the estimates; that essentially this difference was a spurious observation. Consequently, this further underscores the need to understand more about the influence of examiner participation rates on the estimates produced by VESCA.

Whilst the one of the ostensible benefits of Rasch modelling (including Many Facet Rasch Modelling) is its ability to equate from relatively limited linkage patterns [27], very little prior literature has considered the impact of different linking patterns on the validity of its estimates. Consequently, whilst data can theoretically be linked with as little as 7% linkage [18], empirical work is heterogenous and does not give a clear pattern. For example Myford and Wolfe [28] found no consistent relationship between the number of linking performances and the quality of linkage. Conversely, Wind et al. [29] found the strength of linkage deteriorated sequentially with fewer linking performances. Most work has considered individual examiners rather than examiner-cohorts, and none has considered reduced examiner participation rates. Consequently, there is little prior research to guide the implications for VESCA in this scenario.

### Practical implications

These theoretical considerations mean that VESCA should not yet be used to adjust scores in practice, until statistical simulation has supported or refuted their use. Nonetheless, our study has some practical implications. Firstly, it has further shown that videoing in OSCE exams can be feasibly employed. This has several potential uses, including in video feedback to both candidates [30] and examiners or to enable remote examining [31] which may in turn help to aid authenticity by altering the artificial triadic interaction between student, simulated patient and examiner [32]. Moreover, this study has shown that regardless of the validity of score adjustments, VESCA offers a feasible means to compare the scoring of different groups of examiners at different sites within a distributed OSCE. As prior work has suggested substantial geographic variations in expectations within knowledge testing for graduating medical students [33], having a means to compare examiners stringency/leniency across sites within a distributed OSCE is needed to ensure equivalence and is likely to benefit quality assurance of OSCEs. The reasons for comparatively low examiner participation rates in this OSCE are unclear. Anecdotally, this may be because the recent pressures of the COVID-19 pandemic on examiners reduced willingness or capacity to take part in research. Notably examiner participation was much higher in prior uses of VESCA conducted prior to the pandemic [12] but determining longer term engagement will require further prospective investigation. This is being investigated through ongoing research using Realist evaluation, which explores how students and examiners use and interact with VESCA including their reasons for engaging with the intervention or not. This research will also understand students’ opinions relating to score adjustment in a range of circumstances. Regardless, further efforts are needed to understand what measures can be taken to ensure examiners engagement with video scoring to maximise the utility of VESCA for quality assurance purposes.

Any intervention to enhance either reliability or validity of an OSCE must be considered in light of its cost, as these collectively impact the utility of an assessment [34]. Video camera set up and subsequent video processing collective required approximately 12 h of technician time, whilst video scoring required approximately 40 min of time per examiner. Given these resource implications and the need for psychometric expertise, VESCA may find its greatest utility in large scale or national exams, and investigating its utility across multiple locations or institutions in a national context would be informative. Within a single school, where these resources could pose challenges, increasing station numbers or faculty development could prove more cost effective.

### Limitations

The principal limitation of this study are examiner participation rates, which, as stated, limit our ability to interpret students’ score adjustments. Videos were all filmed at one site; whilst videoed students broadly represented the student cohort, it is unknown whether using a mixture of students from both sites would have altered findings. Videos were filmed using volunteer students and examiners, which could potentially have altered their behaviour. Prior work has suggested that students are minimally impacted by carefully placed cameras [13], whilst examiners were aware that their scoring behaviour was not being directly scrutinized. Moreover, videoed students broadly represented the range of performances in the student cohort. Consequently, the impact of these limitations is expected to be small. Analysis relied on the equivalence of scores given to video and live performance. Our data and a range of prior findings support the equivalence of these scores.

### Suggestions for future research

Future research, using simulation, should seek to determine to what extent score adjustments produced by VESCA more accurately represent students’ performances, and how a range of operational parameters (number of linking videos, examiner participation rates, range of students’ abilities) influence these estimates. Further research should explore more about how, when and why examiners will score videos in order to maximise engagement with the intervention.

## Conclusions

This study has, for the first time, used VESCA to compare the influence of fully nested groups of examiners across different sites in a distributed OSCE. Whilst the findings suggest potentially substantial differences in stringency of different groups of examiners, more work is needed to determine the influence of examiner participation and other parameters on the estimates provided. VESCA appears to offer a feasible means to determine the stringency of examiners across different sites in distributed OSCEs which could importantly aid demonstration of equivalence as part of quality assurance work.

## Data Availability

The datasets generated and/or analysed during the current study are not publicly available due the limitations of the study’s ethical approvals but are available from the corresponding author on reasonable request.
